# The Efficacy of Teaching English as a Foreign Language to Iranian Students with Autism Spectrum Disorder on Their Social Skills and Willingness to Communicate

**Published:** 2019

**Authors:** Fatemeh GOLSHAN, Marjan MOINZADEH, Mehri Haddad NARAFSHAN, Mohammad Reza AFARINESH

**Affiliations:** 1 Neuroscience Research Center, Institute of Neuropharmachology, Kerman University of Medical Sciences, Kerman, Iran; 2 California licensed Clinical Psychologist, San Francisco, USA; 3 Department of Foreign Languages, Kerman Branch, Islamic Azad University, Kerman, Iran; 4 Cognitive Research Center, Institute of Neuropharmachology, Kerman University of Medical Sciences, Kerman, Iran

**Keywords:** Autism spectrum disorder, English, Social skills, Willingness to communicate

## Abstract

**Objectives:**

This applied research is the first practical study of teaching English as a foreign language (EFL) to students with autism spectrum disorder (ASD) in Iran. We examined the effect of a well-designed foreign language learning setting in facilitation of social skills and willingness to communicate in children with ASD.

**Materials & Methods:**

A mixed-method research design was used. Using stratified sampling, a limited sample of 18 students were chosen from Kerman Province, southeastern Iran in 2014 categorized in three levels of ASD for each group of experimental and control; matched pairs were used to ensure homogeneity of participants in two groups. Each participant received 15 sessions with totaling 67 h of language learning. First 10 sessions were in the form of tutorials and the last 5 sessions were held in the form of paired classes with a peer. Before and after the sessions, caregivers and parents completed a questionnaire on students' social skills; the English instructor also rated participants' willingness to communicate.

**Results:**

Teaching a foreign language had a positive main effect on social skills from caregivers’ and parents’ view compared to those of controls, significantly (*P*<0.05). From the instructor's view, there was additionally a significant improvement in the students with ASD’s willingness to communicate in classroom settings compared to the control group (*P*<0.05).

**Conclusion:**

Optimum foreign language pedagogy for students with ASD is applied as an effective context enhancing children’s capabilities in social skills and willingness to communicate, provoked through a motivational foreign setting modulation in a novel environment. Suggestions on enhancing joint attention during the curriculum are provided.

## Introduction

Autism spectrum disorder (ASD) is a lifelong neuro-biological disease that has drastically increased with an incompatible rise during the past three decades (1). The main reason for considering autism as a spectrum disorder is due to its variant ranges, the major of which would fluctuate from communication deficiency, e.g. giving an unsuitable response in conversations, misunderstanding nonverbal communications or problems with building up new friendships; to unduly repetitive actions, oversensitivity to environmental changes, or exaggerated focus on irrelevant items (2). The neurobiology of ASD is difficult to define, due to the neurodevelopmental characters, and the large phenotypic heterogeneity of individuals with ASD (3). They often suffer from comorbid disorders, particularly social anxiety disorder, oppositional defiant disorder, and attention-deficit/hyperactivity disorder (4).

Children with autism lack appropriate social skills (SS) which result in restricted social interaction (5). Verbal and non-verbal social behaviors are essential to effectively engage in interpersonal communication. Broadly, these skills can be divided into seven domains including interaction, communication, cooperation, assertion, responsibility, empathy, engagement, and self-control (6).

One of the exceptional cases, when a child with ASD is required to deal with a compulsory conversation to enhance his SS, happens in a language classroom. For children with autism, classroom settings can pose particular challenges when conversing with classmates is expected. Foreign language classrooms present a unique challenge and opportunity; however, because all students will be communicating in a way that is new to them (so all students regardless of ability face some challenges), for children with autism whose language is also impaired the foreign language environment may compound the social obstacles. Yet these environments create possibilities for students with autism. Many pupils with ASD, as less extroverted students, suffer from “difficulties relating to others and presenting their own selves adequately due to their limited communicative competence”, which directly influences their progress (7). One of the fundamental components of instruction for children with autism is to create opportunities for social interaction and for them to practice their social skills. Throughout the past few decades, educational research has advanced immensely in areas such as special education and language learning (8-10).

Willingness to communicate (WTC) was first defined as “an individual’s volitional inclination toward actively engaging in the act of communication in a specific situation, which can vary according to interlocutor (s), topic, and conversational context, among other potential situational variables” (11). This process is tangible as each student is motivated by the class design and expresses signs of WTC in different manners. 

The conceptual framework of this study was the cognitive development approach of Vygotsky. According to Vygotsky's social learning theory, there is a reciprocity between language acquisition and social development. During cognitive development and social learning, a teacher can effectively engage in scaffolding a proper social model for learners and the preliminary of a successful learning environment is to keep their joint attention during learning process (12-15). Many studies have so far focused on the descriptive analysis of fundamental factors required in a language classroom for students with ASD (16-19). There are studies which explore well-rounded environments for foreign language learning by students with ASD; accordingly, our study not activates a well-designed curriculum for students with ASD but also practices on enhancing their social skills through conversation modeling (20, 21).

Despite the public concern about the unexplained growth of ASD (22), there is yet a considerable gap in literature between students with ASD and English language learners (23), including lack of communication and presence of open-minded educators to embrace pedagogical adaptability based on the need of each individual so that it results in successful language learning as well as building communication models to be used in further steps of life (24). 

We aimed to explore how a foreign language learning coursework influences SS in learners with ASD. This provides learning opportunities for students to acquire WTC as well as a new language (25, 26).

## Materials & Methods

**Participants**

Participants were chosen from Imam Ali Autism School in Kerman Province, southeastern Iran in 2014. The 74 students of the school were diagnosed using Gilliam Autism Rating Scale (GARS). The whole population was then divided into three levels of ASD as follows: 

Level 1: Requiring support (high-functioning disorder)

Level 2: Requiring substantial support (mild-functioning disorder)

Level 3: Requiring very substantial support (low-functioning disorder).

By stratified sampling, the 18 out of 74 were chosen with equal numbers (6 members) in each level. Each level was randomly divided into two experimental and control groups (3 members per groups). Consequently, 9 students were assigned to the experimental group and 9 comparable students included in the control group in ensure homologous matched pairs for this study. To certify cause-effect relationship which is crucial for internal validity, the effect of some variables were minimized; the students were matched on the basis of their autism level, age, ethnicity, and domestic background. The inclusion criteria for parents were matched level of education, social and financial status. 

All the students were at the true beginner level of English as a foreign language learning according to Oxford Young learners' placement test; they were all from the middle class with the same social and domestic background in the age range of 8-12. The demographic and clinical information of each participant is present in Table 1.

Both parents and caregivers were included to fill questionnaires and report children's social behaviors, the reason for holding these two groups for answering the questionnaires was because they spent the most amount of time with each individual and therefore had dynamic aspect of individual's socialization. The English instructor as well as a clinical psychologist also observed the process of language courses and verified homogeneity of the syllabus design and the accuracy of reported behaviors.

**Ethical issues**

Before the sessions started, the researchers obtained informed consent from each participant’s parents. All the sessions were recorded and were provided to the parents if required. In the meantime, parents reported children's daily activities at home every day. While in some countries, there is a particular committee like Institutional Review Board (IRB) designated to observe and review all human biomedical and behavioral researches, this study is also reported to be in complete congruence with ethical issues of Neuroscience Research center of Kerman (Grant No: KNRC/EC/94-63). 

Additionally, the special school psychologist had confirmed the validity of class syllabus design in order to conform to each individual’s case and requirements. 

**Instruments**

This study was examined through both questionnaires and direct observation. Examining the quantitative aspect of the study, we focused on two distinct dependent variables: SS and WTC measured via questionnaires. In order to confirm the psychometric properties of the evaluation on a qualitative scale, both translated questionnaires were first evaluated based on professional comments from psychologists and autism specialists. For internal validity, Cronbach alpha was used which assessed construct validity of the research. To examine internal consistency of measurement items (construct validity), Cronbach alpha was 0.87 for SS questionnaire and 0.75 for WTC questionnaire. Moreover, the research design was chosen to be based on stratified sampling and matched levels for experimental and control groups. 

 The first questionnaire was Vanderbilt Kennedy Center’s “Triad Social Skills Assessment” (27) with a reliability of 0.98 and validity of 0.92. This questionnaire was translated into Persian before being distributed between both societies of two psychologist caregivers and parents respectively to collect data on how both groups evaluate children’s general SS. This questionnaire was distributed as a form of pre-test/ post-test two weeks before and after the course. The second questionnaire was designed with a reliability of 0.83 and validity of 0.94. The English WTC questionnaire of this study was designed to reflect the students’ attitude toward EFL learning with the consideration of each individual’s WTC in the foreign language (28). This questionnaire was filled by the language instructor in the form of pre-test/post-test two weeks before and after the course. The experimental group was supplied by a total EFL learning term, while the control group was provided by the same term duration with materials in Farsi. The English courses mainly focused on lexical instruction (e.g. emotions, times of the day, and greetings), the Alphabet jigsaw tasks and English nursery rhymes. For teaching aids, a bear puppet designed by Oxford University (29) was utilized as a facilitator of sensory desensitization to stabilize each student’s attention and progressively amplify the quality of stimuli response. The educational tools of the class design were Picture Exchange Communication System (PECS) and video modeling. Each classroom lesson plan was adjusted to the individual’s preference and circumstances by the clinical psychologist. Before beginning, a school psychologist evaluated the class syllabus for experimental and control classes to ensure continuity of instruction. Not only did we use direct observation for in language classes and outside-classroom social interactions, but also the whole language instruction process was recorded to make more tangible access for parents to reach their children's language class.

 A 67-h exclusive English course was designed to teach students with ASD English. Each student was provided with 15 individual recorded sessions lasting for average 45 min each day. The educational curriculum was confirmed by both the English instructor (mastered in teaching English as a second language) and the clinical psychologist who observed every recorded session of individuals’ language learning. The first 10 sessions were held in the form of language tutorials in order to control distraction and stimulus equivalence during language learning. Each session had modified educational approaches and contents based on special conditions of each individual. After completing 10 sessions, two random students were joined in the same classes for 5 sessions in order to be observed and evaluated on the basis of language use and social interaction. The results were then reflected in WTC questionnaires.

**Data Analysis**

Both *t*-test and paired *t*-test were used to evaluate the quantitative data for significance. In terms of the qualitative data, EFL was analyzed as an independent variable to have effects on students' SS and WTC.

## Results

Before the treatment, there were no statistically significant differences between the experimental and control groups based on their SS and WTC scores. For a better realization of the autism spectrum distinction and behavior, the subjects were classified on the basis of each individual’s GARS into three categories of low, mild, and severe ASD in both experimental and control group.

*What is the influence of teaching English as a foreign language on students with ASD’s SS from the parents and caregivers’ view?*

**Figure 1:**
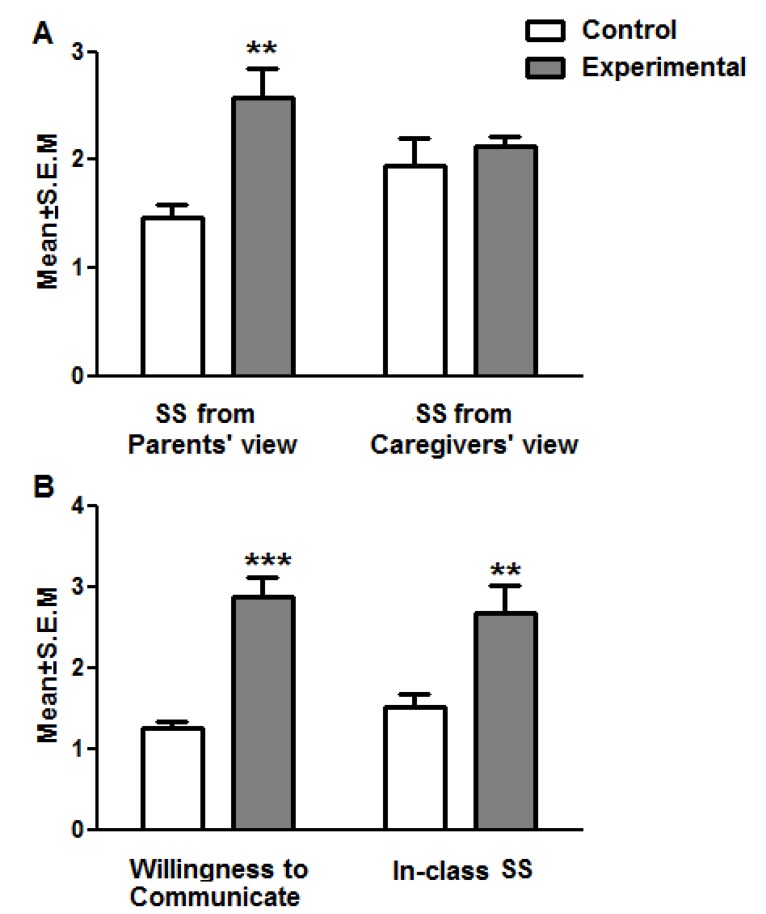
Total comparison between the control (n=9) and experimental (n=9) groups in social skills (SS) from parents and caregivers’ views (A) and willingness to communicate and in class SS (B). The data are Mean±S.E.M. Symbol (*) shows a significant difference between control and experimental groups following a t-tests student statistic analysis. **P<0.01 and ***P<0.001.

SS of students with ASD had a significant increase of about 15% in the experimental group compared to the control group according to the parents’ view (*t*-test, *P*<0.01) (Figure 1). In all three groups, EFL significantly affected the amount of SS from parents’ view (t-test, *P*<0.05, *P*<0.01, and *P*<0.05, respectively) (Table 2).

Despite the fact that the statistical analysis displayed SS to have increased about 10% in students with ASD following EFL learning on the basis of the caregivers’ view, this advancement was not thoroughly significant (*t*-test, *P*>0.05) (Figure 1). Both groups of low and mild ASD had a notable increase in SS (*t*-test *P*<0.05, and *P*<0.001, respectively) (Table 2). 

*What is the influence of teaching EFL on students with ASD’s WTC from the English instructor’s view?*

WTC considerably increased in the experimental group compared to the control group (t-test, *P*<0.001) (Figure 1). Moreover, all three categories of low, mild, and severe ASD had an increase in WTC respectively in the experimental group (*t*-test, *P*<0.001, *P*<0.001, and *P*<0.01) (Table 2).

The effectiveness of teaching EFL on students’ in-class SS was additionally displayed to have a positive advancement in experimental group compared to the control group (t-test, *P*<0.01) (Figure 1) with significance in two categories of low and mild ASD (*t*-test, *P*<0.01, and *P*<0.05, respectively) (Table 2).

## Discussion

We aimed to investigate the relation between foreign language pedagogies and the process of SS and WTC advancement. While one of the fundamental key roles of language classes is to optimize human communication, this potentiality was utilized to facilitate social interactions for students with ASD. 

Some of the students could have suffered from language disorders, such as expressive aphasia, or individual mental conditions, they were initially offered some tutorial EFL courses specifically designed to adjust their potentials and morphological requirements in the process of language learning. They were subsequently provided with shared classes with a random-paired student with ASD in order to maximize their social interaction in a language-learning environment. As the most critical essence in educational goals, it was more important to provoke their either verbal or nonverbal endeavor to initiate or maintain SS.

There was a notable advancement in SS of students who acquired EFL compared to the control group who were taught in their mother tongue. Our study also showed that, in the experimental group, students with ASD had a more meaningful advancement in their SS according to their parents' views compared to caregivers' views. To discuss this finding, caregivers were responsible for controlling classes including 20 students with ASD caused caregivers' share of attention among all the students in the classroom than careful focusing on each individual's way of communication. Parents, however, reported every detail of social reactions made in children's daily routines inside and outside home since they spent their whole time with the child. Moreover, most of the socialization signs mainly reveal from domestic environment where the child feels quite supported indeed. Therefore, more meticulous observation of the parents on each individual's advancements could have caused greater notice of the progress compared to the caregivers.

**Table 1:** Demographic description of Students in the Control (A-I) and Experimental (a-i) groups

**Cases**	**Age**	**GARS Score**	**Hyper(+),** **hypo (-) sensitivity**	**Language disorder**	**Mild retardation**	**Moderate retardation**	**Severe retardation**	**Severe** **Inattention disorder**	**Social ** **Communication disorder**	**ADHD**	**Self-injuring** ** Behavior**
**A**	11	83 /ST 0.23	-	-				*			
**B**	8	87/ST 0.25	+	+aphasia					*	*	
**C**	12	63/ST 0.10	-	-				*	*		
**D**	8	91/ST 0.27	-	+aphasia	*			*			
**E **	12	63/ST 0.19	+	-	*			*		*	*
**F**	10	63/ST 0.22	+	-		*			*		
**G**	10	101/ST 0.31	+	-			*		*		*
**H **	12	118/ST 0.31	-	-		*		*	*		
**I**	8	117/ST 0.38	-	+			*	*			
**a**	11	68/ST 0.24	-	-				*	*		
**b**	10	61/ST 0.31	-	-				*			
**c**	8	58/ST 0.21	+	-	*					*	
**d**	8	88/ST 0.12	+	+aphasia	*				*		
**e**	10	92/ST 0.23	+	-		*		*		*	*
**f**	12	103/ST 0.39	-	-		*		*	*		
**g**	8	107/ST 0.28	+	-		*					
**h**	12	109/ST 0.10	-	-			*	*	*		*
**i**	12	115/ST 0.19	-	+	*				*		*

**Table 2:** Pre-test and post-test results of both control and experimental group

**Variables**		**Ctl**		**Exp**
Social skills from parents view	1.high-functioning	Pre-Test	1.95±0.15	3.15±0.58
		Post-Test	1.84±0.21	3.35±0.44*
	2.mild-functioning	Pre-test	1.35±0.096	4.05±2.50
		Post-Test	3.60±2.71	###4.12±2.49**
	3.low-functioning	Pre-Test	1.32±0.19	2.39±0.68
		Post-Test	1.73±0.75	2.37±0.81*
Social skills from caregivers’ view	1.high-functioning	Pre-test	2.86±0.31	2.13±0.19
		Post-Test	2.86±0.29	2.20±0.11*
	2.mild-functioning	Pre-test	1.64±1.10	3.84±2.61
		Post-test	3.90±2.56	3.80±2.64***
	3.low-functioning	Pre-test	1.39±0.27	1.82±0.78
		Post-Test	2.12±0.80	1.79±0.83
Willingness to communicate	1.high-functioning	Pre-test	1.2±0.38	1.13±0.08
		Post-Test	1.37±0.21	###3.42±0.19***
	2.mild-functioning	Pre-Test	1.08±0.02	3.40±2.81
		Post-Test	3.50±2.76	#4.19±2.47***
	3.low-functioning	Pre-test	1.17±0.11	1.42±0.83
		Post-Test	1.58±0.76	2.25±0.96*
In-class social communication	1.high-functioning	Pre-Test	1.95±0.04	1.76±0.12
		Post-Test	2.09±0.09	#3.61±0.40**
	2.mild-functioning	Pre-test	1.14±0.08	3.69±2.66
		Post-Test	3.67±2.68	4.23±2.43*
	3.low-functioning	Pre-Test	1.28±0.21	1.66±0.80
		Post-Test	1.75±0.83	2.44±0.88

In addition, the students’ WTC in English was also described with a significant rise after attending English classes according to the English instructor's view. The children were reported to have demonstrated sponteneaous social initiation in English with the instructor and their paired peer (in the last five sessions). Most social initiations were performed in the form of basic greetings in English accompanied by body language (such as waving hands).

 The main impact on SS and WTC advancement was demonstrated in level 2 of ASD (mild-functioning); hence since there was a lower amount of socialization in this group compared to level 1 (high-functioning), the efficacy of language lessons on improving SS and WTC could have been projected more meaningfully. Since individuals with ASD in level 3 (low-functioning) suffered from a more diversity of complicated conditions, less significant progress was shown in their SS or WTC. No effect of other features such as language disorder, e.g. expressive aphasia was reported on children’s results of SS or WTC in this study.

To explain the qualitative result of English classes, all the verbal students in level 1 and 2 of ASD from the experimental group achieved a basic amount of both receptive and productive results in language acquisition. However, the amount of each individual’s acquired materials was mainly influenced by their GARS scores and mental conditions. As the most significant phenomenon of the present study, one of the students with selective mutism and expressive aphasia in the experimental group (based in level 2) managed to commence basic verbal expressions and consequently shared a significant amount of SS with the peers and caregivers both in mother tongue and English. He was reported to have started studying at a mainstream school with a zeal for studying English 9 months after the study. 

Our findings implicate future studies in three main fields: 1. More practices on the integration of foreign language pedagogies and the curriculum for training students with ASD; 2. The role of language learning approaches and tools in joint attention; and 3. The study of neuropsychological perspective in the relation between socialization and language acquisition.

First, despite more recent encouragement by the governmental entities increased the reported evidence-based practices for children with ASD due to their importance as specific set of interventions implemented by educators (30), more single-subject efforts may be a setback in the field. Various criteria such as the broad range of difficulties or comorbidities in each case, different environmental considerations can change the efficacy of a single method for each child; this is why it is crucial to acknowledge the type of article to be informative about methods to teach English to students with ASD rather than didactic (31). By the influx of English necessity as a global language for each nation, the main focus of this study was not only to teach EFL to students with ASD as an educational purpose; but it was also determined to probe English teaching as an intervention targeting children’s SS provoked through an interesting foreign setting modulation in a language classroom. With the concerning rise of ASD, covering more educational or therapeutic answers is a priority. Students with ASD’s EFL learning interest and capability necessitate attaining required standards through a close association of specialists to cover both psychological and educational gaps for optimizing language-learning environment. In developing countries such as Iran, the deprivation of children with ASD from optimized language classes is quite tangible since those with normal IQ cannot be included in mainstream language classes and there are no professionals to be expert in both domains of language teaching knowledge and ASD treatments. Therefore, as the implication of the current study, any further investigations which signify the alliance of both special needs (such as in ASD) and EFL situational constraints is truly auspicious. 

In addition, from a behavioral perspective, since English language has never been utilized in any aspect of the students’ daily routines in Iran, the peculiarity of sounds and expressions, culture-oriented body gestures and expressive behaviors for students with ASD has acted as a motivator to remain in the communication and achieve moderate attention during social practices. One of the initial attempts of each facilitator or trainer is to draw the student’s attention on the exercises and keep the eye contact, using an English nursery rhyme, to exemplify, can be a quite more practical stimulus than usual nursery rhymes in the mother tongue to reinforce attention. Therefore, attention which acts as the key factor for social initiation and maintenance for students with ASD can be assumed as a prospective subject to be investigated during the process of language learning in future studies.

Finally, to explain the relevant neurophysiological function of the brain, the adjacent interconnection between vocalization and communication is suggested to be the role of the right Superior Temporal Sulcus (STS) of the brain (32). This part is mainly accountable for dyad of responsibilities including both the language processing activity and social attention e.g. eye gaze processing in order to signify communicative inputs (33-37). The dual tasks of this region, i.e. activating in response to communicative signals such as biological motions as well as speech, raise the possibility of STS to act as the main responsible region for distinguishing communicative vocal sounds compared with the normal non-communicative ones such as a sneeze or a cough per se (38). Hence, this anatomical substrate signifies the close reciprocal association of speech perception and social acquisition (39). This could seemingly explain the voice processing problems in social communication disorders as in autism spectrum disorder since STS as a voice-selective region is believed to function hyperactively in response to vocal sounds and lack preferential attention to speech (40). Knowing this neurophysiological significance of STS in both auditory and visual inputs e.g. body movements, head orientation, lip reading, and facial expression (39, 41) can help to describe why communicative functions with a different vocal processing stimuli represented in teaching a foreign language can engage more social interaction in the present study. New communicative vocal sounds of a foreign language could gradually provoke a rise in cortical voice processing and therefore, lead to a leap in the interpersonal communication; this hypothetical issue would, therefore, require further studies.

**In conclusion, **as there is a rapid rise in the population of children with autism, the need to motivate higher standards in education requires an interdisciplinary approach including not only the teachers or trainers but also psychologists and speech therapists to monitor the process.
